# Evaluation of the Cleveland Clinic Score for predicting acute kidney injury across different elective cardiac surgeries—a retrospective study

**DOI:** 10.7717/peerj.20533

**Published:** 2026-01-15

**Authors:** Mateusz Kozioł, Vladyslav Kyslyi, Dorota Sobczyk, Jacek Piatek, Janusz Konstanty-Kalandyk

**Affiliations:** 1 Medical College, Jagiellonian University, Krakow, Poland; 2 Department of Cardiovascular Surgery and Transplantology, John Paul 2nd Hospital, Jagiellonian University, Krakow, Poland

**Keywords:** Acute kidney injury, Cardiac surgery, Cleveland clinic score, Acute kidney injury prediction model

## Abstract

**Background:**

Acute kidney injury (AKI) after cardiac surgery is a serious postoperative complication associated with an increased risk of mortality. The Cleveland Clinic Score (CCS) is one of the tools that allows preoperative assessment of the likelihood of developing AKI. However, the tool has not been validated in different types of cardiac surgery procedures. Our aim was to evaluate the CCS before different types of cardiac surgery and to assess the usefulness of this tool as a predictor of AKI.

**Methods:**

In this retrospective study we included patients who underwent elective cardiac surgery in 2023. Our endpoint was AKI, as defined by the Kidney Diseases Improving Global Outcomes (KDIGO) criteria. The predictive value for AKI after cardiac surgery (CCS) was evaluated using receiver operating characteristic (ROC) curves and area under the curve (AUC) values.

**Results:**

A total of 610 patients underwent elective cardiac surgery. Patients with and without AKI were divided into CCS stages: stage I (57.8 *vs* 72.3%), stage II (36.1 *vs* 26.1%), stage III (5.4 *vs* 1.6%), stage IV (0.6 *vs* 0%). The AUC for all operations was 0.630 (95% CI [0.580–0.679], *p* < 0.001), stage I 0.428 (95% CI [0.376–0.480]; *p* = 0.006), stage II 0.550 (95% CI [0.498–0.602]; *p* = 0.057) and for stage III 0.519 (95% CI [0.467–0.572]; *p* = 0.464). The AUC values were significant only for coronary artery bypass grafting (CABG) 0.650 (95% CI [0.552–0.748]) and aortic valve replacement/plasty (AVR/AVP) 0.629 (95% CI [0.550–0.709]).

**Conclusions:**

The overall CCS value showed a moderate predictive ability for AKI (AUC 0.630) and particularly useful for predicting renal replacement therapy (RRT), but for individual groups the scale should be modified by adding several new factors.

## Introduction

Acute kidney injury (AKI) is a serious complication that can occur after cardiac surgery. Depending on the author and definition used, 5–30% of cardiac surgery patients may develop AKI. Exposure to nephrotoxic drugs and substances, such as aminoglycosides and radiocontrast agents, may increase the risk of developing AKI (Chertow et al., 1997; Rosner & Okusa, 2006; Huen & Parikh, 2012; Bhat et al., 1976; Gailunas et al., 1980; Corwin et al., 1989). The reported pathophysiological mechanisms for AKI caused by cardiac surgery include ischemic injury, renal reperfusion syndrome, inflammation, atheroembolism, neurohormonal activation, and oxidative stress (Sethi et al., 1987; Perella et al., 1992; Wan, LeClere & Vincent, 1997; Doty et al., 2003). The risk of mortality associated with AKI after open heart surgery is 5 times higher in patients with AKI than in patients without AKI (Thakar et al., 2007; Rao et al., 2018; Korczak et al., 2022).

The use of less invasive cardiac surgical techniques and off-pump coronary artery bypass grafting has resulted in lower mortality rates and incidences of acute renal failure among patients undergoing minimally invasive and/or off-pump surgery. However, post-operative renal dysfunction has remained unchanged in conservative open-heart surgery. After cardiac surgery, one to five percent of patients require dialysis for AKI, and this condition is strongly associated with perioperative morbidity and mortality (Zhang et al., 2008; Swaminathan et al., 2007). The mortality rate of patients with AKI requiring dialysis is estimated to be greater than 50% (Lopez-Delgado et al., 2013). AKI associated with cardiac surgery increases the risk of infection and prolongs hospital and intensive care unit stays (Bove et al., 2004; Lassnigg et al., 2004). This increases the use of healthcare resources and is associated with higher mortality (Ryckwart et al., 2002).

Several risk models have been developed to estimate the risk of postoperative kidney injury after cardiac surgery. Among these models, the Cleveland Clinic model is the most widely tested, and according to several studies, it has the highest discriminative power in most populations tested  (Huen & Parikh, 2012; Englberger et al., 2010). The Cleveland Clinic Score (CCS), developed by Thakar et al. (2005), is a clinical tool to predict AKI risk after cardiac surgery, incorporating factors such as female gender, congestive heart failure, left ventricular ejection fraction (LVEF) <35%, preoperative intra-aortic balloon pump (IABP) use, chronic obstructive pulmonary disease (COPD), insulin-dependent diabetes, previous cardiac surgery, emergency surgery, type of surgery, and preoperative creatinine levels, with scores ranging from 0 to 17 points (Table 1). It can be used in the preoperative evaluation of AKI and is appropriate for all patients undergoing cardiac surgery. Predicting the development of AKI, its early treatment and prevention are goals of cardiac surgeons and nephrologists involved in the care of these patients, providing an opportunity to develop strategies for early diagnosis and treatment. Recent studies have proposed enhancing the CCS by incorporating additional predictors such as baseline hemoglobin, estimated glomerular filtration rate (eGFR), and glycosylated hemoglobin (HbA1c), which may improve its predictive accuracy across diverse patient populations (Vives et al., 2024).

**Table 1:** Components of the Cleveland Clinic score.

Parameter	Weight
Female gender	1
Congestive heart failure	1
LVEF <35%	1
Preoperative IABP	2
COPD	1
Diabetes with insulin	1
Previous cardiac surgery	1
Emergency surgery	2
Surgery type	
CABG	0
Valve only	1
CABG + Valve	2
Other cardiac surgeries	2
Preoperative creatinine	
<1.2 mg/dl	0
1.2 to <2.1 mg/dl	2
≥2.1 mg/dl	5
Addition of points, minimum score 0, maximum 17	
Points Risk of dialysis	
0–2 0.4%	
3–5 1.8%	
6–8 7.8–9.5%	
9–13 21.5%	

**Notes.**

Abbreviations CABG coronary artery bypass grafting COPD chronic obstructive pulmonary disease IABP intra-aortic balloon pump LVEF left ventricular ejection fraction

The purpose of the study was to evaluate the Cleveland Clinic Score before different types of surgery and to assess its usefulness as a predictor of acute kidney injury after elective cardiac surgery.

## Materials & Methods

### Study design and participants

In this retrospective study, we enrolled 610 patients aged 18 years and older who underwent cardiac surgery between January and December 2023. For this retrospective study, we enrolled 610 patients aged 18 years and older who underwent cardiac surgery between January and December of 2023. We excluded patients who were younger than 18 years old, those who underwent non-elective surgery, and those whose type of surgery was performed fewer than 10 times during this period. If a patient underwent more than one cardiac surgery during the same hospitalization, only the data from the first surgery were considered.

We reviewed the databases and medical records of enrolled patients, collecting the data on their demographic details and medical histories, including important clinical, operative and peri-operative data, such left ventricular ejection fraction (LVEF), pre- and post-operative serum creatinine level, estimated glomerular filtration rate (eGFR), European System for Cardiac Operative Risk Evaluation (EuroSCORE), type of surgery, postoperative complications and treatment, and CCS. The CCS was calculated retrospectively for each patient based on preoperative data extracted from medical records, using the standard formula outlined by Thakar et al. (2005) (Table 1).

We could not distinguish between type 1 and type 2 diabetes, so we grouped all diabetic patients into a single category. Because our study was limited to patients with elective surgery, we excluded emergency surgery as a parameter in the Syntax Clinic Score.

Our endpoint was AKI as defined by the Kidney Diseases Improving Global Outcomes (KDIGO) criteria. AKI stage 1 was identified by an increase in serum creatinine of ≥ 0.3 mg/dL (≥26.4 µmol/L) or an increase of 1.5–1.9 times baseline. AKI stage 2 was defined by an increase in serum creatinine of 2–2.9 times baseline. AKI stage 3 was defined by a 3-fold increase in baseline serum creatinine, a serum creatinine increase to four mg/dL (353.6 µmol/L), or the need for renal replacement therapy (RRT). Due to data limitations and the potential effect of postoperative diuretic use, urine output criteria were not included in the KDIGO definition of AKI. This may have led to underdiagnosis of AKI, particularly in cases where urine output was reduced without significant creatinine changes, as urine output is a sensitive early indicator of AKI.

### Ethics

This study was approved by the Research Ethics Committee of the Medical College of Jagiellonian University (approval number: 118.0043.1.119.2024) and was conducted in accordance with the Declaration of Helsinki. The written informed consent was obtained from the participants upon their admission to the hospital.

### Statistical analysis

In this retrospective study, an inferential statistical analysis was performed using SPSS Statistics V29.0 (IBM Corp., Armonk, NY, USA). Categorical variables were compared between groups using Fisher’s exact test or the chi-2 test and presented as counts and percentages. Differences in continuous variables were tested with the Mann–Whitney U test, presented as median along with interquartile range (IQR) or with the t-Student test, presented as mean with standard deviation (SD).

The test characteristics (sensitivity, specificity, positive and negative predictive values) of CCS as a predictor of AKI were evaluated by constructing ROC curves and calculating the corresponding area under the curve (AUC) values. Statistical significance was determined at a *p*-value of less than 0.05.

## Results

Between January and December of 2023, a total of 610 patients were admitted for elective surgery (see Table 2). Of those patients, AKI of any stage occurred in 166 patients (27.2%) after elective cardiac surgery. Patients without AKI had fewer comorbidities, such as arterial hypertension, diabetes mellitus, chronic kidney injury, atrial fibrillation, and carotid artery disease, compared with patients with AKI. They were also significantly younger (65 *vs.* 69 years, *p* < 0.001) and had a higher estimated glomerular filtration rate (eGFR). There were no significant differences between the AKI and non-AKI groups with respect to sex, chronic pulmonary disease, preoperative anemia, coronary or peripheral artery disease, history of stroke, smoking status, body mass index, left ventricular ejection fraction, or type of surgery. Patients with elevated baseline serum creatinine levels and a higher EuroSCORE II score were more likely to develop AKI after cardiac surgery (*p* < 0.001).

**Table 2:** Baseline characteristics for patients with and without postoperative AKI.

Parameter	Total (*n* = 610)	AKI (*n* = 166)	No AKI (*n* = 444)	*p*-value
Age, median (IQR)	66 (60–71)	69 (64–74)	65 (58–70)	<0.001
Age >65, n (%)	331 (54.3)	115 (69.3)	216 (48.6)	<0.001
Age >70, n (%)	185 (30.3)	72 (43.4)	113 (25.5)	<0.001
Sex, n (% male)	450 (73.8)	117 (70.5)	333 (75.0)	0.259
Asthma/COPD, n (%)	80 (13.1)	21 (12.7)	59 (13.3)	0.836
Obstructive sleep apnea, n (%)	39 (6.4)	11 (6.6)	28 (6.3)	0.886
Arterial hypertension, n (%)	522 (85.6)	155 (93.4)	367 (82.7)	<0.001
Diabetes mellitus, n (%)	203 (33.3)	68 (41.0)	135 (30.4)	0.014
Impaired Glucose Tolerance, n (%)	60 (9.8)	24 (14.5)	36 (8.1)	0.019
With insulin treatment, n (%)	26 (4.3)	9 (5.4)	17 (3.8)	0.386
Chronic kidney injury, n (%)	59 (9.7)	39 (23.5)	20 (4.5)	<0.001
Dialysis, n (%)	7 (1.1)	7 (4.2)	0	<0.001
Other kidney diseases (cyst, nephrolithiasis), n (%)	70 (11.5)	22 (13.3)	48 (10.8)	0.400
Atrial fibrillation, n (%)	73 (12.0)	31 (18.7)	42 (9.5)	0.002
Anemia, n (%)	22 (3.6)	5 (3.0)	17 (3.8)	0.630
Coronary artery disease, n (%)	321 (52.6)	94 (56.6)	227 (51.1)	0.226
Carotid Artery Disease, n (%)	51 (8.4)	20 (12.0)	31 (7.0)	0.044
Peripheral artery disease, n (%)	35 (5.7)	12 (7.2)	23 (5.2)	0.333
Stroke, n (%)	42 (6.9)	16 (9.6)	26 (5.9)	0.101
Smoking history, n (%)	121 (19.8)	32 (19.3)	89 (20.0)	0.832
pCO2 (mmHg), mean (SD)	37.308 (3.5751)	37.505 (3.5170)	37.235 (3.5977)	0.406
pO2 (mmHg), median (IQR)	89.4 (81–100)	88.7 (81.9–98.9)	89.55 (80.85–100)	0.980
Weight (kg), median (IQR)	82 (73–92)	83 (73–93)	82 (73–92)	0.742
BMI (kg/m^2^), median (IQR)	28.405 (25.6–31.53)	28.88 (26.45–31.72)	28.295 (25.39–31.415)	0.081
BMI >30 kg/m^2^, n (%)	216 (35.4)	65 (39.2)	151 (34.0)	0.267
LVEF (%), median (IQR)	60 (50–60)	58 (50–60)	60 (50–60)	0.267
LVEF =<35, n (%)	34 (5.6)	14 (8.4)	20 (4.5)	0.060
Creatinine before surgery (μmol/l), median (IQR)	82 (72–94)	87 (77–105)	81 (72.0–91.5)	<0.001
Creatinine maximum level (μmol/l), median (IQR)	95 (80–118)	152.5 (122–204)	87 (74–99)	<0.001
eGFR (ml/min/1.73m2), mean (SD)	81.797 (20.39345)	65.89 (21.182)	87.74 (16.565)	<0.001
EuroSCORE, median (IQR)	1.17 (0.82–1.62)	1.425 (1.12–2.32)	1.07 (0.755–1.44)	<0.001
CABG, n (%)	184 (30.2)	44 (26.5)	140 (31.5)	0.229
CABG+AVR, n (%)	35 (5.7)	13 (7.8)	22 (5.0)	0.174
CABG+CAS, n (%)	10 (1.6)	5 (3.0)	5 (1.1)	0.103
MIDCAB, n (%)	52 (8.5)	9 (5.4)	43 (9.7)	0.093
AVR/AVP, n (%)	216 (35.4)	66 (39.8)	150 (33.8)	0.170
AVR/AVP+reoperation, n (%)	15 (2.5)	3 (1.8)	12 (2.7)	0.525
AVR/AVP+LAAO, n (%)	15 (2.5)	3 (1.8)	12 (2.7)	0.525
MVR/MVP, n (%)	29 (4.8)	9 (5.4)	20 (4.5)	0.636
Bental de Bono, n (%)	54 (8.9)	14 (8.4)	40 (9.0)	0.824
Length of intensive care unit stay (days), median (IQR)	1 (1–3)	2 (1–4)	1 (1–3)	<0.001
Length of hospital stay (days), median (IQR)	7 (5–10)	7 (5–13)	6 (5–9)	0.002
Return to intensive care unit, n (%)	20 (3.3)	16 (9.6)	4 (0.9)	<0.001
Transfusions on intensive care unit, n (%)	378 (62.0)	125 (75.3)	253 (57.0)	<0.001
Rethoracotomy, n (%)	48 (7.9)	26 (15.7)	22 (5.0)	<0.001
Pulmonary complications, n (%)	114 (18.7)	54 (32.5)	60 (13.5)	<0.001
Respiratory failure, n (%)	18 (3.0)	14 (8.4)	4 (0.9)	<0.001
NIV, n (%)	47 (7.7)	21 (12.7)	26 (5.9)	0.005
Pleural fluid, n (%)	58 (9.5)	32 (19.3)	26 (5.9)	<0.001
Reintubation, n (%)	14 (2.3)	14 (8.4)	0	<0.001
Tracheotomy, n (%)	4 (0.7)	4 (2.4)	0	0.001
Pneumonia, n (%)	37 (6.1)	21 (12.7)	16 (3.6)	<0.001
Wound infection, n (%)	49 (8.0)	27 (16.3)	22 (5.0)	<0.001
VAC/drainage/re-suturing, n (%)	22 (3.6)	12 (7.2)	10 (2.3)	0.003
CVVHD, n (%)	17 (2.8)	15 (9.0)	2 (0.5)	<0.001
Readmission, n (%)	31 (5.1)	19 (11.4)	12 (2.7)	<0.001
In-hospital death, n (%)	18 (3.0)	10 (6.0)	8 (1.8)	0.006
Post-operative inotropic support, n (%)	160 (26.2)	62 (37.3)	98 (22.1)	<0.001
Cleveland Clinic Score, median (IQR)	2 (1–3)	2 (2–3)	2 (1–3)	<0.001
Stage I (scores 0–2), n (%)	417 (68.4)	96 (57.8)	321 (72.3)	<0.001
Stage II (scores 3–5), n (%)	176 (28.9)	60 (36.1)	116 (26.1)	0.015
Stage III (scores 6–8), n (%)	16 (2.6)	9 (5.4)	7 (1.6)	0.008
Stage IV (scores 9–13), n (%)	1 (0.2)	1 (0.6)	0	0.102

**Notes.**

*P* value is presented for the comparison of AKI-group with no-AKI group. Values are mean (SD), n (%), or median (interquartile range).

Abbreviations ASD/PFO atrial septal defect/patent foramen ovale AVR/AVP aortic valve replacement/plasty BMI body mass index CABG coronary artery bypass graft CAS carotid artery stenting COPD chronic obstructive pulmonary disease; Creatinine level reference range –52-106 μmol/l CVVHD continuous veno-venous hemodialysis eGFR estimated glomerular filtration rate (normal range >60) EuroSCORE European System for Cardiac Operative Risk Evaluation IQR interquartile range LAAO left atrial appendage occlusion LVEF left ventricular ejection fraction MIDCAB minimally invasive direct coronary artery bypass MVR/MVP mitral valve replacement/plasty NIV non-invasive ventilation SD standard deviation SI conversion factor for eGFR to ml/s - 0.0167 TVP tricuspid valve plasty VAC vacuum-assisted closure

During the postoperative period, re-thoracotomy, the need for blood transfusions in the ICU, and the need for intravenous inotropic support were more frequent in the AKI group.

Patients with AKI after cardiac surgery, were also more likely to develop postoperative pulmonary complications (including respiratory failure, pleural fluid and pneumonia) and surgical site infection (including deep wound infection necessitating drainage and resuturing). They also spent more time in the hospital (both ICU and postoperative clinic) and had higher hospital mortality. Continuous veno-venous hemodialysis (CVVHD) was used in 15 patients (9%) with AKI and in 2 (0.5%) without AKI (*p* < 0.001). In patients without AKI, CVVHD has been used for the management of peripheral fluid retention due to postoperative left ventricular failure.

**Figure 1:**
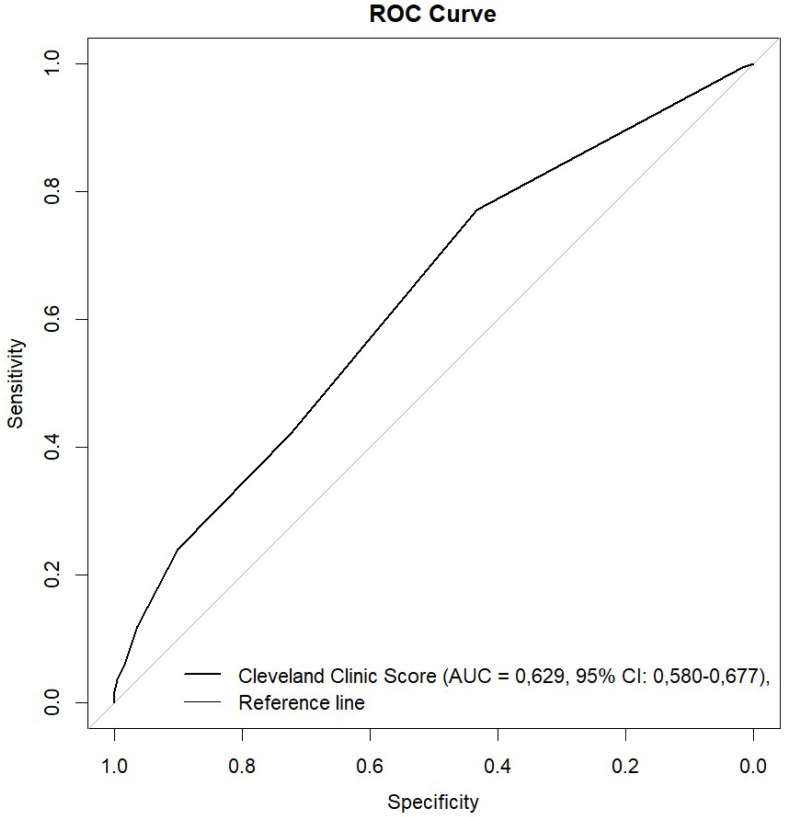
ROC curve.

**Figure 2:**
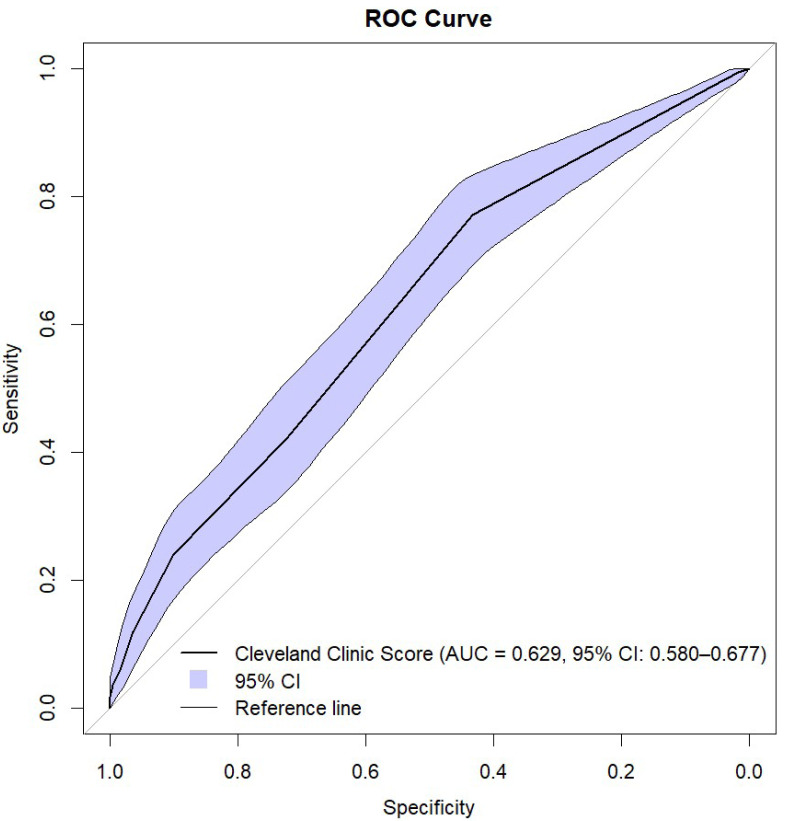
ROC curve with bootstrap analysis.

**Figure 3:**
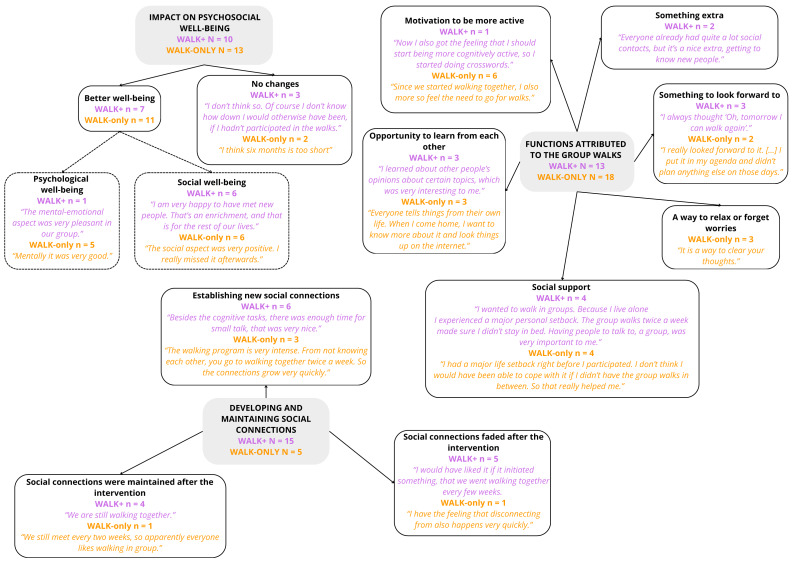
ROC curves for different surgery types.

The Cleveland Clinic Scores were significantly higher in the AKI group. Using ROC analysis, CCS showed predictive ability for AKI with need for RRT in all patients with an AUC of 0.630 (95% CI [0.580–0.679]; *p* ≤ 0.001) (Figs. 1 and 2, Table 3). The results indicate that the Cleveland Clinic score has a high sensitivity of over 70%, with a relatively low specificity of over 40%. This means that it detects the disease with high sensitivity in most patients, but may produce false-positive results in healthy individuals. This is a valuable clinical observation, as it means that the diagnosis of an increased risk of AKI based on CCS requires additional confirmation with other tests and laboratory results. The values were statistically significant for CABG (AUC 0.650; 95% CI [0.552–0.748]; *p* = 0.003) and aortic valve replacement/plasty (AVR/AVP) (AUC 0.629; 95% CI [0.550–0.709]; *p* = 0.002). In the case of predictive analysis for CABG and AVR/AVP, for which statistical significance was demonstrated, sensitivity and specificity were 63%, 60% and 75%, 48%, respectively. CCS was predictive for AKI stages II (AUC 0.550; *p* = 0.057) and III (AUC 0.519; *p* = 0.464) (Fig. 3, Table 4). The better performance of CCS for CABG and AVR/AVP may be due to shared pathophysiological mechanisms, such as atherosclerosis in coronary artery disease and aortic stenosis, which are captured by CCS components like preoperative creatinine and LVEF (Table 1).

## Discussion

The true incidence of acute kidney injury (AKI) in patients undergoing cardiac surgery is unclear because different authors have used different terminology to define it. In a South Asian cohort of 276 patients, the overall incidence of AKI, as defined by the KDIGO criteria, was 6.88% (Rao et al., 2018). Wong et al. (2015) reported AKI in 14.5% of patients after cardiac surgery. Robert et al. (2010) analyzed a group of 25,086 patients undergoing cardiac surgery in northern New England. According to the Acute Kidney Injury Network (AKIN) criteria, AKI occurred in 30% of patients, and according to the Risk, Injury, Failure, Loss, and End-stage kidney disease (RIFLE) criteria, it occurred in 31%. In our study, acute kidney injury of any stage occurred in 27.2% of patients after elective cardiac surgery according to the KDIGO criteria.

In our study, we identified the following preoperative risk factors for AKI development: greater number of comorbidities, older age, and higher European System for Cardiac Operative Risk Evaluation (EuroSCORE) II score. EuroSCORE is a cardiac risk model that predicts 30-day mortality after cardiac surgery. Derived from an international European database, it was first introduced in 1999 and updated to its second version, EuroSCORE II, in 2011 (Nashef et al., 2012).

Preoperative renal impairment has been proven to be an independent risk factor for postoperative mortality. Therefore, creatinine clearance (CCr) was incorporated into EuroSCORE II with three levels of renal insufficiency: moderate (CCr 50–85 mL/min), severe (<50 mL/min), and on dialysis (irrespective of CCr). Thus, elevated EuroSCORE values in AKI patients may reflect reduced preoperative renal function. AKI patients were also more likely to undergo rethoracotomy, receive blood transfusions, and require inotropic support during the perioperative period. All of these postoperative factors are associated with transient renal ischemia. It has been shown that early postoperative AKI after cardiac surgery is strongly associated with two major factors: reduced functional reserve and renal ischemia.

AKI is a frequent complication following cardiac surgery, increasing the risk of mortality, morbidity, prolonged hospital stays, and hospital costs. Identifying patients at high risk for postoperative kidney damage following cardiac surgery is the first step in preventing this complication. Several scoring models have been developed to facilitate risk stratification and improve clinical decision-making (Pannu et al., 2016; Alhulaibi et al., 2022; Chen et al., 2016; Jiang et al., 2017; Vogt et al., 2021; Dedemoğlu & Tüysüz, 2020; Elmedany et al., 2017).

In our study, we used the CCS, a prognostic tool that assesses the risk of postoperative acute kidney injury. However, it should be emphasized that both the Cleveland Clinic score and the Euroscore depend heavily on preoperative serum creatinine levels, which are the most influential factor. The Canadian study by Wong et al. (2015) evaluated the CCS’s ability to predict both AKI requiring dialysis and less severe stages of AKI in 2,316 patients from a tertiary care center. The study found that the CCS was valid in identifying patients with severe stages of AKI but had less discriminative power for earlier stages.

**Table 3:** Area under the curve values of different types of surgeries.

Surgery	AUC ROC CCS	95% Cl	*p*-value
CABG	0.650	0.552–0.748	0.003
CABG+AVR/AVP	0.680	0.489–0.871	0.079
CABG+CAS	0.820	0.553–1.000	0.095
MIDCAB	0.602	0.386–0.818	0.339
AVR/AVP	0.629	0.550–0.709	0.002
AVR/AVP+ reoperation	0.722	0.419–1.000	0.248
AVR/AVP+LAAO	0.667	0.284–1.000	0.386
MVR/MVP	0.681	0.467–0.894	0.126
Bental de Bono	0.468	0.281–0.654	0.722
All surgery	0.630	0.580–0.679	<0.001

**Notes.**

Abbreviations AUC area under curve AVR/AVP aortic valve replacement/plasty CABG coronary artery bypass grafting CAS carotid artery stenting CCS Cleveland Clinic Score LAAO left atrial appendage occlusion MIDCAB minimally invasive direct coronary artery bypass MVR/MVP mitral valve replacement/plasty

Our novel approach was to apply this score to the general cardiac surgical population and evaluate its usefulness with respect to different types of surgery (*e.g.*, CABG, valve surgery). Our analysis suggests that using the CCS to estimate risk by type of surgery may provide more accurate AKI predictions. Most studies have focused on the general use of the CCS score without considering the diversity of surgical procedures, and only a few have differentiated CCS scores for specific types of surgery, such as coronary artery bypass grafting (CABG), valve surgery, and other surgeries (Huen & Parikh, 2012).

In our analysis, for the entire study, the AUC ROC CCS was 0.63 (0.580−0.679; 95% CI).

Additionally, we confirmed these results in a bootstrap analysis. When analyzing the clinical usefulness of the test, we assessed that Cleveland Clinic Score has a high sensitivity of over 70%, with a relatively low specificity of over 40%. This means that it detects the disease with high sensitivity in most patients, but may produce false-positive results in healthy individuals.

Our approach revealed that different types of surgery are associated with distinct risk profiles that the CCS can effectively capture with the proper adjustments. ROC analysis demonstrated the statistical power to predict AKI in patients undergoing CABG (AUC 0.650; *p* = 0.003) and aortic valve replacement/repair (AVR/AVP) (AUC 0.629; *p* = 0.002) (Fig. 1, Table 3). The pathophysiological mechanisms of AKI after cardiac surgery include ischemia and atheroembolism. Atherosclerosis is the common pathological basis of coronary artery disease and aortic stenosis. This may explain the importance of the CCS in predicting AKI in patients undergoing CABG and aortic valve surgery. However, it should be noted that the small group size may limit the reliability of these findings.

**Table 4:** Area under the curve values of different types of surgeries divided into Cleveland Clinic Score stages.

Surgery	AUC ROC CCS 1	95% Cl	*p*-value	AUC ROC CCS 2	95% Cl	*p*-value	AUC ROC CCS 3	95% Cl	*p*-value
CABG	0.411	0.310–0.512	0.075	0.558	0.458–0.659	0.243	0.531	0.430–0.631	0.542
CABG+AVR/AVP	0.477	0.279–0.676	0.824	0.484	0.282–0.686	0.878	0.538	0.335–0.742	0.707
CABG+CAS	0.500	0.125–0.875	1.000	0.300	0.000–0.644	0.296	0.700	0.356–1.000	0.296
MIDCAB	0.380	0.158–0.601	0.261	0.620	0.399–0.842	0.261	0.500	0.291–0.709	1.000
AVR/AVP	0.440	0.355–0.526	0.162	0.562	0.477–0.648	0.146	0.498	0.414–0.581	0.955
AVR/AVP+ reoperation	0.500	0.123–0.877	1.000	0.375	0.000–0.771	0.516	0.625	0.229–1.000	0.516
AVR/AVP+LAAO	0.375	0.014–0.736	0.516	0.625	0.264–0.986	0.516	0.500	0.123–0.877	1.000
MVR/MVP	0.403	0.171–0.634	0.409	0.542	0.309–0.775	0.724	0.500	0.269–0.731	1.000
Bental de Bono	0.561	0.385–0.736	0.502	0.429	0.255–0.602	0.430	0.511	0.332–0.690	0.906
All surgery	0.428	0.376–0.480	0.006	0.550	0.498–0.602	0.057	0.519	0.467–0.572	0.464

**Notes.**

Abbreviations AVR/AVP aortic valve replacement/plasty AUC area under curve CABG coronary artery bypass grafting CAS carotid artery stenting CCS Cleveland Clinic Score LAAO left atrial appendage occlusion MIDCAB minimally invasive direct coronary artery bypass MVR/MVP mitral valve replacement/plasty

Many studies propose including additional factors in the scale, such as urine biomarkers (*e.g.*, neutrophil gelatinase-associated lipocalin, interleukin-6, hepcidin-25, and midkine), age, body mass index (BMI), and the incidence of arrhythmia and pulmonary hypertension.; serum markers such as lactate, total bilirubin, albumin, white blood cells, cystatin C, and uric acid; cardiopulmonary bypass time; aortic cross-clamp duration; and central venous pressure (Albert et al., 2020; Yan et al., 2023; Huang et al., 2022; Wang et al., 2022; Che et al., 2019; Palomba et al., 2007; Krzanowska et al., 2024). Introducing these factors into clinical practice could lead to better risk management and improved patient outcomes during surgical procedures.

CCS is primarily used to evaluate the risk of postoperative RRT. In our study, we found that CCS grades 2 and 3 values are just above the reference line, indicating an increased risk of RRT. However, grade 1 is well below this line. These results align with the CCS scale’s assumptions, confirming that the total score and individual stages are effective prognostic tools, particularly for identifying patients requiring postoperative RRT.

It should be noted, however, that further studies are needed to fully verify the usefulness and accuracy of the CCS in different surgical contexts. Therefore, our results may serve as a foundation for future studies aimed at refining the CCS scale to meet the unique requirements of various surgical procedures. Our findings suggest that clinicians can use the CCS scale to identify patients at higher risk for acute kidney injury requiring renal replacement therapy, particularly for coronary artery bypass grafting and aortic valve replacement/aortic valve plasty, enabling targeted preoperative optimization. For milder AKI stages or other surgeries, however, alternative or more refined risk models may be necessary.

### Study limitations

Our analysis has several limitations. First, it is a retrospective, single-center study. Second, we included a limited number of patients. Third, we only used creatinine criteria and did not include urine criteria due to data limitations and the postoperative diuretic effect.

## Conclusions

Our study found that the Cleveland Clinic Score was a moderate predictor of acute kidney injury and was especially useful in identifying patients who needed renal replacement therapy, especially those undergoing CABG and AVR/AVP procedures. According to our findings, the predictive accuracy of the CCS may be enhanced for different stages of AKI and cardiac surgery types by taking into account variables like advanced age, preoperative estimated glomerular filtration rate, and comorbidities like diabetes and hypertension, which demonstrated strong correlations with AKI.

##  Supplemental Information

10.7717/peerj.20533/supp-1 Supplemental Information 1 Raw data

10.7717/peerj.20533/supp-2 Supplemental Information 2 Codebook for raw data

## Additional Information and Declarations

### Data Availability

The following information was supplied regarding data availability: The raw data is available in the Supplemental File.

## References

[ref-1] Albert C, Haase M, Albert A, Kropf S, Bellomo R, Westphal S, Westerman M, Braun-Dullaeus RC, Haase-Fielitz A (2020). Urinary biomarkers may complement the cleveland score for prediction of adverse kidney events after cardiac surgery: a pilot study. Annals of Laboratory Medicine.

[ref-2] Alhulaibi AA, Alruwaili AM, Alotaibi AS, Alshakhs FN, Alramadhan HS, Koudieh MS (2022). Validation of various prediction scores for cardiac surgery-associated acute kidney injury. Journal of the Saudi Heart Association.

[ref-3] Bhat JG, Gluck MC, Lowenstein J, Baldwin DS (1976). Renal failure after open heart surgery. Annals of Internal Medicine.

[ref-4] Bove T, Calabro MG, Landoni G, Aletti G, Marino G, Crescenzi G, Rosica C, Zangrillo A (2004). The incidence and risk of acute renal failure after cardiac surgery. Journal of Cardiothoracic and Vascular Anesthesia.

[ref-5] Che M, Wang X, Liu S, Xie B, Xue S, Yan Y, Zhu M, Lu R, Qian J, Ni Z, Zhang W, Wang B (2019). A clinical score to predict severe acute kidney injury in chinese patients after cardiac surgery. Nephron.

[ref-6] Chen J, Zhang G, Wang C, Liu Y, Han L, Lu F, Xu Z (2016). Predicting renal replacement therapy after cardiac valve surgery: external validation and comparison of two clinical scores. Interdisciplinary CardioVascular and Thoracic Surgery.

[ref-7] Chertow GM, Lazarus JM, Christiansen CL, Cook EF, Hammermeister KE, Grover F, Daley J (1997). Preoperative renal risk stratification. Circulation.

[ref-8] Corwin HL, Sprague SM, De Laria GA, Norusis MJ (1989). Acute renal failure associated with cardiac operations. Journal of Thoracic and Cardiovascular Surgery.

[ref-9] Dedemoğlu M, Tüysüz ME (2020). Risk estimation model for acute kidney injury defined by KDIGO classification after heart valve replacement surgery. General Thoracic and Cardiovascular Surgery.

[ref-10] Doty JR, Wilentz RE, Salazar JD, Hruban RH, Cameron DE (2003). Atheroembolism in cardiac surgery. Annals of Thoracic Surgery.

[ref-11] Elmedany SM, Naga SS, Elsharkawy R, Mahrous RS, Elnaggar AI (2017). Novel urinary biomarkers and the early detection of acute kidney injury after open cardiac surgeries. Journal of Critical Care.

[ref-12] Englberger L, Suri RM, Li Z, Dearani JA, Park SJ, Sundt 3rd TM, Schaff HV (2010). Validation of clinical scores predicting severe acute kidney injury after cardiac surgery. American Journal of Kidney Diseases.

[ref-13] Gailunas P, Chawla R, Lazarus JM, Cohn L, Sanders J, Merrill JP (1980). Acute renal failure following cardiac operations. Journal of Thoracic and Cardiovascular Surgery.

[ref-14] Huang T, He W, Xie Y, Lv W, Li Y, Li H, Huang J, Huang J, Chen Y, Guo Q, Wang J (2022). A LASSO-derived clinical score to predict severe acute kidney injury in the cardiac surgery recovery unit: a large retrospective cohort study using the MIMIC database. BMJ Open.

[ref-15] Huen SC, Parikh CR (2012). Predicting acute kidney injury after cardiac surgery: a systematic review. Annals of Thoracic Surgery.

[ref-16] Jiang W, Xu J, Shen B, Wang C, Teng J, Ding X (2017). Validation of four prediction scores for cardiac surgery-associated acute kidney injury in chinese patients. Brazilian Journal of Cardiovascular Surgery.

[ref-17] Korczak A, Morawiec R, Stegienta M, Ryk A, Walczak A, Krekora J, Krejca M, Drozdz J (2022). Acute kidney injury as the most important predictor of poor prognosis after interventional treatment for aortic stenosis. Kardiologia Polska.

[ref-18] Krzanowska K, Batko K, Niezabitowska K, Woźnica K, Grodzicki T, Małecki M, Bociąga-Jasik M, Rajzer M, Sładek K, Wizner B, Biecek P, Krzanowski M (2024). Predicting acute kidney injury onset with a random forest algorithm using electronic medical records of COVID-19 patients: the CRACoV-AKI model. Polish Archives of Internal Medicine.

[ref-19] Lassnigg A, Schmidlin D, Mouhieddine M, Bachmann LM, Druml W, Bauer P, Hiesmayr M (2004). Minimal changes of serum creatinine predict prognosis in patients after cardiothoracic surgery: a prospective cohort study. Journal of the American Society of Nephrology.

[ref-20] Lopez-Delgado JC, Esteve F, Torrado H, Rodríguez-Castro D, Carrio ML, Farrero E, Javierre C, Ventura JL, Manez R (2013). Influence of acute kidney injury on short and long–term outcomes in patients undergoing cardiac surgery: risk factors and prognostic value of a modified RIFLE classification. Critical Care.

[ref-21] Nashef SAM, Roques F, Sharples LD, Nilsson J, Smith C, Goldstone AR, Lockowandt U (2012). Euroscore II. European Journal of Cardio-Thoracic Surgery.

[ref-22] Palomba H, De Castro I, Neto AL, Lage S, Yu L (2007). Acute kidney injury prediction following elective cardiac surgery: AKICS score. Kidney International.

[ref-23] Pannu N, Graham M, Klarenbach S, Meyer S, Kieser T, Hemmelgarn B, Ye F, James M, Approach Investigators and the Alberta Kidney Disease Network (2016). A new model to predict acute kidney injury requiring renal replacement therapy after cardiac surgery. Canadian Medical Association Journal/Journal de L’Association Medicale Canadienne.

[ref-24] Perella MA, Edell ES, Krowka MJ, Cortese DA, Burnett Jr JC (1992). Endothelium—derived relaxing factor in pulmonary and renal circulation during hypoxia. American Journal of Physiology.

[ref-25] Rao SN, Shenoy MP, Gopalakrishnan M, Kiran BA (2018). Applicability of the Cleveland clinic scoring system for the risk prediction of acute kidney injury after cardiac surgery in a South Asian cohort. Indian Heart Journal.

[ref-26] Robert AM, Kramer RS, Dacey LJ, Charlesworth DC, Leavitt BJ, Helm RE, Hernandez F, Sardella GL, Frumiento C, Likosky DS, Brown JR, Northern New England Cardiovascular Disease Study Group (2010). Cardiac surgery-associated acute kidney injury: a comparison of two consensus criteria. Annals of Thoracic Surgery.

[ref-27] Rosner MH, Okusa MD (2006). Acute kidney injury associated with cardiac surgery. Clinical Journal of the American Society of Nephrology.

[ref-28] Ryckwart F, Boccara G, Frappier JM, Colson PH (2002). Incidence: risk factors and prognosis of a moderate increase in plasma creatinine early after cardiac surgery. Critical Care Medicine.

[ref-29] Sethi GK, Miller DC, Souchek J, Oprian C, Henderson WG, Hassan Z, Folland E, Khuri S, Scott SM, Burchfiel C (1987). Clinical, hemodynamic, and angiographic predictors of operative mortality in patients undergoing single valve replacement. Journal of Thoracic and Cardiovascular Surgery.

[ref-30] Swaminathan M, Shaw AD, Phillips-Bute BG, McGugan-Clark PL, Archer LE, Talbert S, Milano CA, Patel UD, Stafford-Smith M (2007). Trends in acute renal failure associated with coronary artery bypass graft surgery in the United States. Critical Care Medicine.

[ref-31] Thakar CV, Arrigain S, Worley S, Yared JP, Paganini EP (2005). A clinical score to predict acute renal failure after cardiac surgery. Journal of the American Society of Nephrology.

[ref-32] Thakar CV, Worley S, Arrigain S, Yared JP, Paganini EP (2007). Improved survival in acute kidney injury after cardiac surgery. American Journal of Kidney Diseases.

[ref-33] Vives M, Candela A, Monedero P, Tamayo E, Hernández A, Wijeysundera DN, Nagore D, Spanish Perioperative Cardiac Surgery Research Group (2024). Improving the performance of the Cleveland Clinic Score for predicting acute kidney injury after cardiac surgery: a prospective multicenter cohort study. Minerva Anestesiologica.

[ref-34] Vogt F, Zibert J, Bahovec A, Pollari F, Sirch J, Fittkau M, Bertsch T, Czerny M, Santarpino G, Fischlein T, Kalisnik JM (2021). Improved creatinine-based early detection of acute kidney injury after cardiac surgery. Interdisciplinary CardioVascular and Thoracic Surgery.

[ref-35] Wan S, LeClere JL, Vincent JL (1997). Inflammatory response to cardiopulmonary bypass: mechanisms involved and possible therapeutic strategies. Chest.

[ref-36] Wang X, Guo N, Chen Y, Dai H (2022). A new model to predict acute kidney injury after cardiac surgery in patients with renal insufficiency. Renal Failure.

[ref-37] Wong B, St Onge J, Korkola S, Prasad B (2015). Validating a scoring tool to predict acute kidney injury (AKI) following cardiac surgery. Canadian Journal of Kidney Health and Disease.

[ref-38] Yan Y, Gong H, Hu J, Wu D, Zheng Z, Wang L, Lei C (2023). Perioperative parameters-based prediction model for acute kidney injury in Chinese population following valvular surgery. Frontiers in Cardiovascular Medicine.

[ref-39] Zhang WF, Gu TX, Diao C, Zhang YH, Wang C, Fang Q, Wang HL (2008). Comparison of transient changes in renal function between off-pump and on-pump coronary artery bypass grafting. Chinese Medical Journal.

